# Narciclasine improves outcome in sepsis among neonatal rats via inhibition of calprotectin and alleviating inflammatory responses

**DOI:** 10.1038/s41598-020-59716-7

**Published:** 2020-02-19

**Authors:** Manoj Kumar Kingsley, Ballambattu Vishnu Bhat, Bhawana Ashok Badhe, Benet Bosco Dhas, Subhash Chandra Parija

**Affiliations:** 1 Department of Neonatology, JIPMER, Puducherry, India; 2 Department of Pathology, JIPMER, Puducherry, India; 3 Department of Microbiology, JIPMER, Puducherry, India

**Keywords:** Neonatal sepsis, Sepsis

## Abstract

Sepsis is associated with exacerbated inflammatory response which subsequently results in multiple organ dysfunction. Sepsis accounts for high mortality and morbidity among newborns worldwide. Narciclasine is a plant alkaloid which has shown to possess anti-inflammatory properties. In this study we investigated the effect and mechanism of action of narciclasine in neonatal sepsis rat models. The excessive release of S100A8/A9 or calprotectin in neonatal sepsis could be detrimental as it could exacerbate the inflammatory responses. We found that narciclasine significantly reduced the plasma levels of S100A8/A9 and also suppressed its expression in the liver and lung. The systemic and local bacterial load was also reduced in the narciclasine treated rats. The systemic and local production of pro-inflammatory cytokines in plasma and organs (liver and lungs) was significantly reduced in the narciclasine treated rats. The histopathological studies showed that narciclasine prevents the organ damage associated with sepsis and improved the survival of neonatal rats. Sepsis increased the phosphorylated NF-κβ p65 protein expression in the liver. Narciclasine suppressed the phosphorylation of NF-κβ p65 and the degradation of NF-κβ inhibitory protein alpha. It could also suppress the expression of adaptor proteins of the toll like receptor signaling pathway viz., myeloid differentiation factor 88 (MyD88), Interleukin-1 receptor-associated kinase 1 (IRAK1) and TNF receptor associated factor 6 (TRAF6). These results suggest that narciclasine protects against sepsis in neonatal rats through the inhibition of calprotectin, pro-inflammatory cytokines and suppression of NF-κβ signaling pathway.

## Introduction

Infection is the most important cause of morbidity and mortality in intensive care units worldwide. As the immune response to infection during the neonatal period is mostly immature it is characterized by high rates of morbidity and mortality^1–3^. Most adults are usually able to restrict the bacterial infection whereas neonates are not able to restrict the bacterial infection and develop a severe systemic inflammatory response. Sepsis is a life threatening health condition which occurs due to the severe and systemic inflammatory response to infection. This syndrome starts with dysregulated inflammation, systemic inflammatory response syndrome (SIRS) progressing to severe sepsis and septic shock^4^. It is the most common cause of death in critically ill patients in non-coronary intensive care units^5^.

Despite the advancement of medical care, sepsis is one of the leading causes of morbidity and mortality among babies in the neonatal intensive care units^6^. This is mainly due to the non-specific signs and symptoms of neonatal sepsis^2^. Antibiotics and supportive care are the mainstay of treatment at present. The nosocomial pathogens are increasingly becoming resistant to the antimicrobials currently being used. Thus alternative therapeutic options such as anti-inflammatory drugs could be beneficial as neonatal sepsis is characterized by elevated levels of inflammatory cytokines such as tumor necrosis factor alpha (TNF-α) and interleukin 6 (IL-6) which amplifies the severity of sepsis^7^. The outburst of inflammatory cytokines in sepsis leads to subsequent complications which finally may end up in multiple organ dysfunction and death. Thus anti-inflammatory drugs could be beneficial in reducing the severity of neonatal sepsis.

Calprotectin is an acute phase reactant present in the cytoplasm of innate immune cells (predominantly neutrophils) and is released immediately after host-pathogen interaction^8^. It is a 24 kda heterodimer consisting of light and heavy chains – MRP 8 and MRP 14 (8 and 14 Kda). In the new S100 protein nomenclature these two units are designated as S100A8 and S100A9 respectively and together they are also known as calprotectin^9,10^. Calprotectin has been proposed for the diagnosis of many inflammatory conditions^11^. Calprotectin is found to be elevated in the circulation during neonatal sepsis and its diagnostic accuracy has been found to be better than other traditional markers^3^. Calprotectin has shown to exhibit cell growth inhibitory, cytotoxic and apoptosis inducing properties on normal cells such as fibroblasts^12–14^.

Plant derived natural products and their derivatives are an invaluable source of therapeutic agents^15,16^. The plants from the Amarylldiaceae family are known for their pharmacologically active alkaloids^17^. The extracts from the bulbs of *Hemanthus coccineus* has been traditionally used as herbal remedy in inflammation associated diseases^18,19^. Narciclasine is an isocarbostyril alkaloid found in the bulbs of *Haemanthus coccineus* extracts and it has been found to exhibit anti-inflammatory properties^20^. Narciclasine has also shown to inhibit the cytotoxicity of calprotectin in rat adjuvant arthritis model^21^. Based on these data we decided to study the potential of narciclasine in inhibiting calprotectin and reducing the excessive inflammatory response in neonatal sepsis. In this study we induced sepsis in neonatal rats using *Escherichia coli* as the infecting agent, as *E. coli* is one of the leading causes of neonatal sepsis^22,23^. We investigated the protective effects of narciclasine in neonatal sepsis rat models and explored the possible mechanisms of action.

## Results

### Narciclasine improved survival in neonatal rats with sepsis

**Figure 1:**
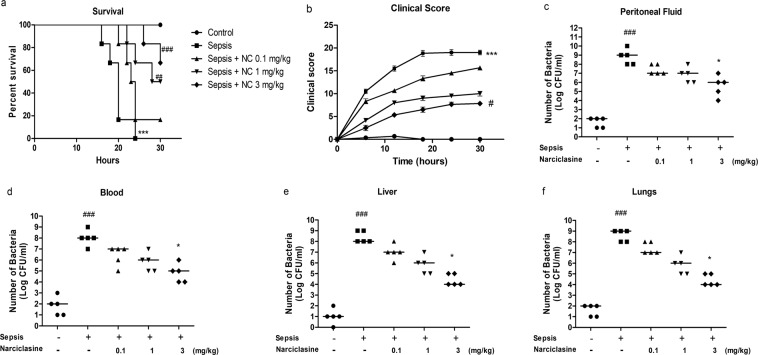
Narciclasine improved survival and reduced the bacterial load in neonatal rats with sepsis. (**a**) Sepsis was induced in the rats (n = 6 per group) followed by administration of narciclasine (NC, 0.1, 1 and 3 mg/kg) after 1 hour and their survival was monitored for 30 hours. Data are presented as the survival percentages of rats. (**b**) The clinical signs were scored for 30 hours after sepsis induction. Data were expressed as mean ± SEM. (**c**–**f**) Sepsis was induced in the rats (n = 6 per group) followed by narciclasine administration (NC, 0.1, 1 and 3 mg/kg) after 1 hour. Blood, peritoneal fluid, livers and lungs were collected after 12 hours. Bacterial counts were estimated after 24 hours of incubation. Data were expressed as medians and compared using Kruskal-Wallis test. (a,b) ***P < 0.001, compared with sham/control group; ^#^P < 0.05, ^##^P < 0.01, ^###^P < 0.001, compared with untreated sepsis group. (c–f) ^###^P < 0.001, compared with sham/control group; *P < 0.05 compared with untreated sepsis group.

Narciclasine treatment improved survival in neonatal rats with sepsis (Fig. 1a). All the rats in the control group survived during the 30 hour observation period. All the rats in the untreated sepsis group were found to be dead before the end of the observation period. The overall survival percentage was 16.67%, 50% and 66.67% on treatment with narciclasine at 0.1 mg/kg, 1 mg/kg and 3 mg/kg respectively at the end of the study period. The untreated sepsis group rats showed a strong rise in the clinical score with progression of time. Narciclasine treatment at 0.1 mg/kg body weight could slightly delay the clinical symptoms, though it was not significant. Treatment with 1 mg/kg showed relatively lower clinical score though not significant compared with the untreated sepsis group. Treatment with 3 mg/kg narciclasine showed minimum clinical symptoms of sepsis and showed a significantly lower clinical score compared with the untreated sepsis group (Fig. 1b). These results suggest that narciclasine improves the survival of neonatal rats with sepsis and relieves of the clinical signs of sepsis.

### Narciclasine reduced the bacterial load in neonatal rats with sepsis

When bacterial dissemination spreads beyond a local environment in the body, the infection and the inflammatory response aimed at restricting the spread of pathogens becomes systemic. This is the scenario in sepsis that subsequently results in organ injury and shock^24^. So restricting the local bacterial load and its spread have significant impact in controlling the pathogenesis of sepsis. We compared the CFU of bacteria in blood, peritoneal fluid, liver and lungs of the various groups. The narciclasine treated rats showed reduction of bacterial load in blood, peritoneal fluid, liver and lungs compared to the sepsis group without treatment, but significant reduction was found only in the 3 mg/kg dosage group (Fig. 1c–f). These data suggest that the beneficial effect of narciclasine in neonatal septic rats could also be due to the reduction of bacterial load at the site of infection and subsequent prevention of systemic bacterial dissemination.

### Narciclasine treatment reduced the plasma levels of inflammatory cytokines in neonatal rats with sepsis

**Figure 2:**
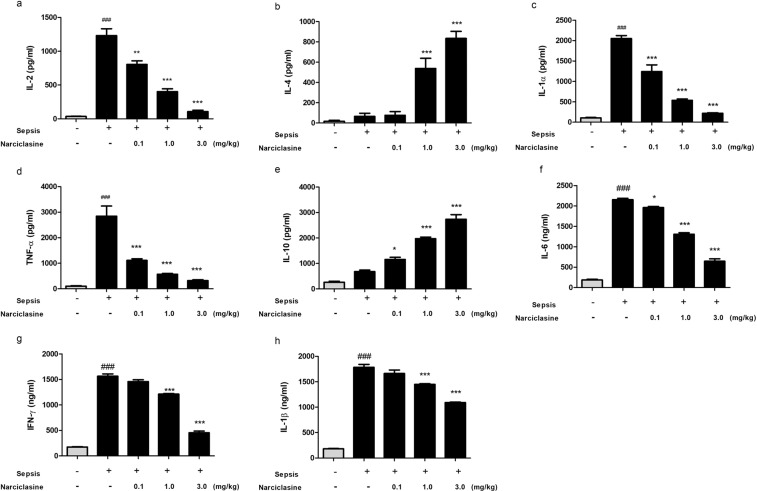
Narciclasine reduced the systemic levels of inflammatory cytokines in neonatal septic rats. The neonatal rats were subjected to *E. coli* induced sepsis (n = 6 per group) in the presence or absence of narciclasine (NC, 0.1, 1 and 3 mg/kg) which was administered 1 hour after sepsis induction. Plasma was separated from blood collected at 12 hours after sepsis induction. (**a**–**e**) The levels of cytokines IL-2, IL-4, IL-1α, TNF-α and IL-10 in plasma were determined by cytometric bead array (**f**–**h**) The levels of cytokines IL-6, IFN-γ and IL-1β in plasma were determined by ELISA. Data were expressed as means ± SEM and compared using ANOVA. ^###^P < 0.001, compared with sham/control group; *P < 0.05, **P < 0.01, ^***^P < 0.001, compared with untreated sepsis group.

Multiple cytokines in plasma were quantified using Cytometric Bead Array (CBA) rat inflammation kit and ELISA. Acquisition by flow cytometry was done using the FACS Calibur II. The results obtained showed that the rats in the untreated sepsis group showed significantly elevated levels of pro-inflammatory cytokines (IL-1α, IL-2, TNF-α, IL-6, IFN-γ, IL-1β) in plasma (Fig. 2a,c,d,f–h). The narciclasine treatment group showed significantly reduced levels of pro-inflammatory cytokines. The levels of anti-inflammatory cytokines (IL-4 and IL-10) in plasma were found to be increased in the narciclasine treatment group compared to the untreated sepsis group (Fig. 2b,e).

### Narciclasine inhibited the expression of S100A8 and S100A9 in septic neonatal rats

**Figure 3:**
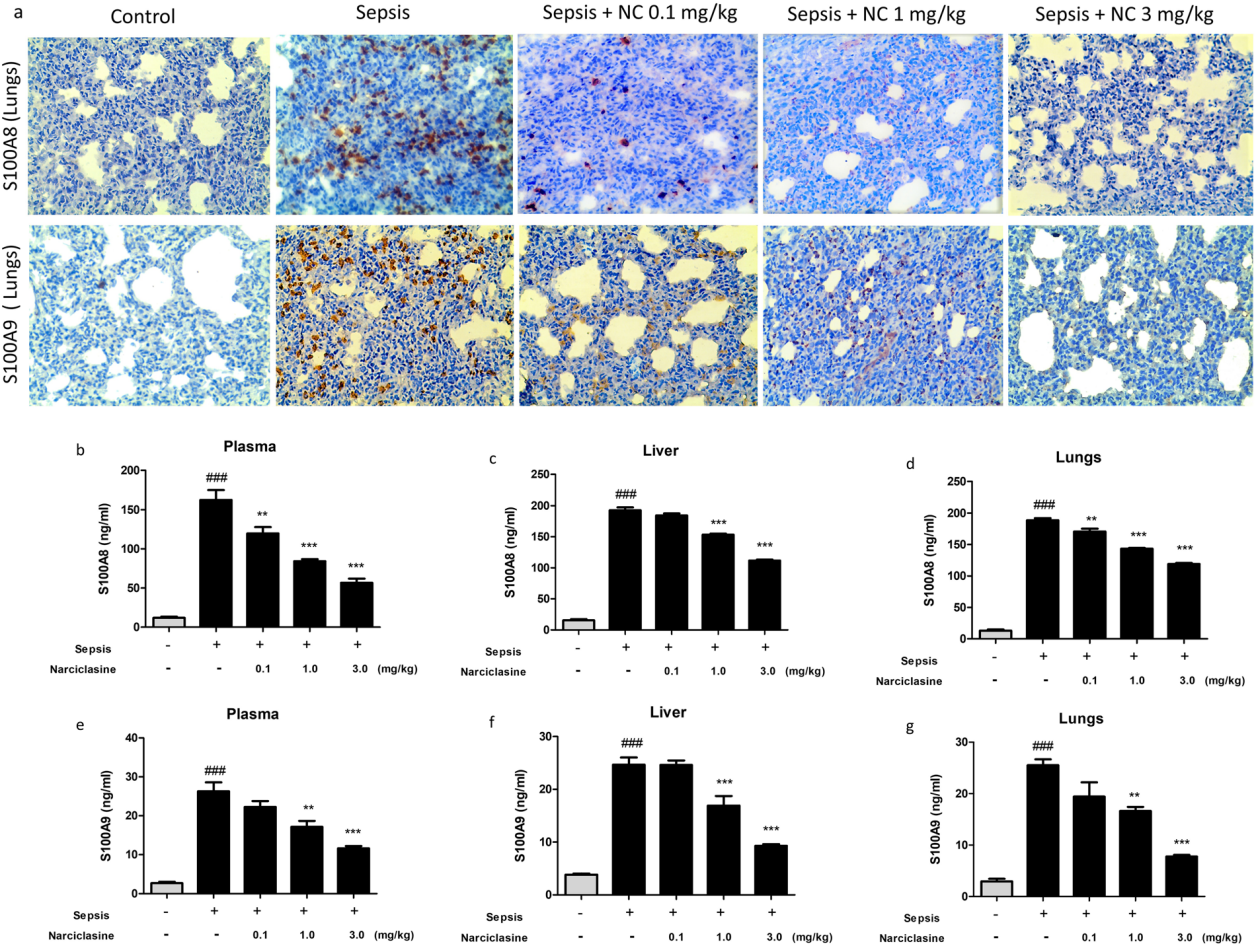
Narciclasine reduced the S100A8 and S100A9 levels in plasma and organ homogenates of neonatal rats with sepsis. The neonatal rats were subjected to *E. coli* sepsis (n = 6 per group) in the presence or absence of narciclasine (NC, 0.1, 1 and 3 mg/kg) which was administered 1 hour after sepsis induction. Plasma, livers and lungs were collected 12 hours after sepsis induction. (**a**) The expression of S100A8 and S100A9 in the lungs was determined by Immunohistochemistry. Amplification x200. The results shown are representative of three independent experiments. The levels of S100A8 in the (**b**) plasma, (**c**) livers and (**d**) lungs were determined by ELISA. The levels of S100A9 in the (**e**) plasma, (**f**) livers and (**g**) lungs were determined by ELISA. Data were expressed as means ± SEM and compared using ANOVA. ^###^P < 0.001, compared with sham/control group; *P < 0.05, **P < 0.01, ***P < 0.001, compared with untreated sepsis group.

**Figure 4:**
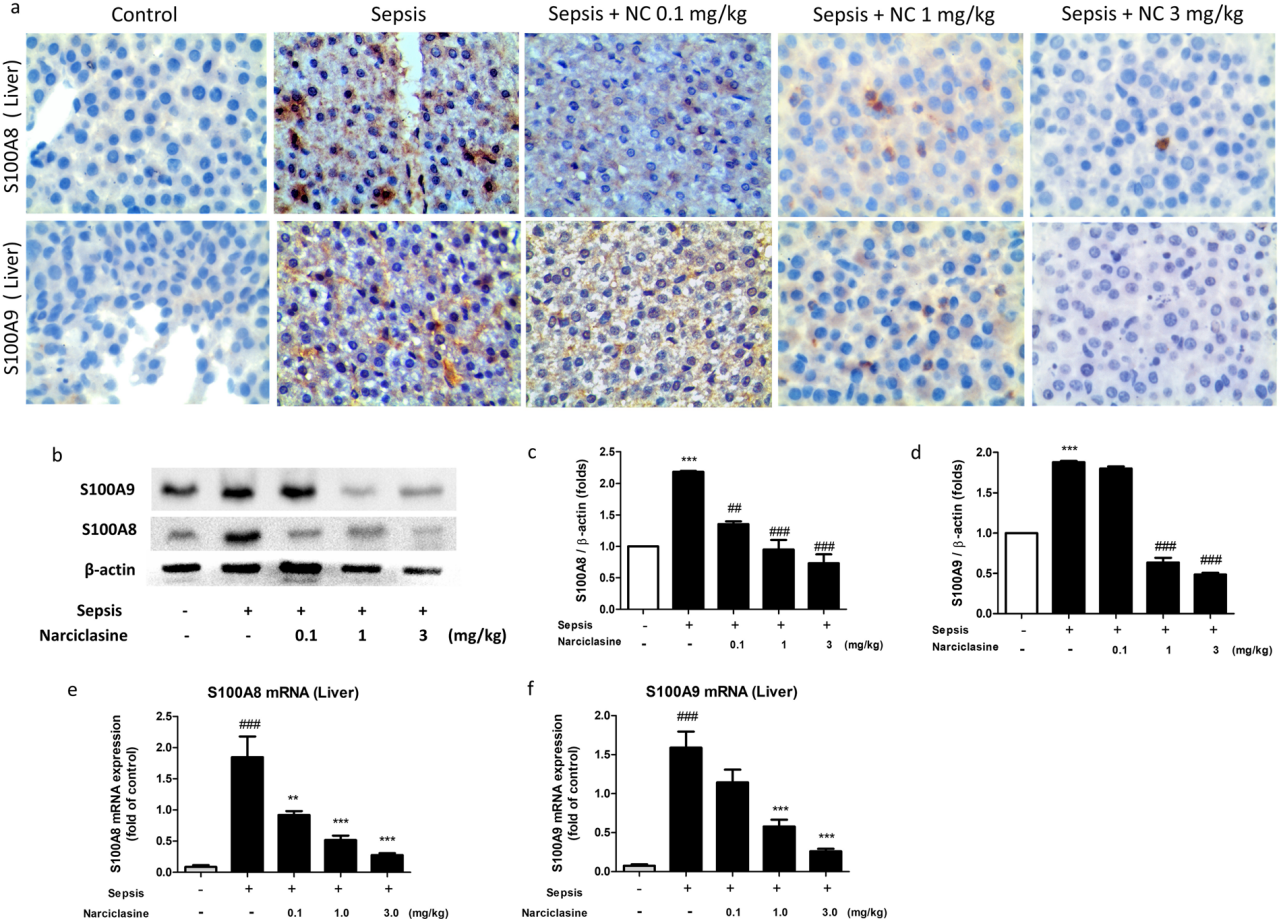
Narciclasine inhibited the expression of S100A8 and S100A9 in the livers of septic neonatal rats. The neonatal rats were subjected to *E. coli* sepsis and narciclasine (NC, 0.1, 1 and 3 mg/kg) was administered 1 hour after sepsis induction. Livers were collected 12 hours after sepsis induction. (**a**) The expression of S100A8 and S100A9 in the livers was determined by Immunohistochemistry. Amplification x400. (**b**) The protein expression of S100A8 and S100A9 in the liver was determined by western blot. The relative levels of (**c**) S100A8 *vs* β-actin, (**d**) S100A9 vs β-actin were determined by densitometry. The mRNA expression of (**e**) S100A8 and (**f**) S100A9 in the liver was determined by quantitative real time PCR. Data were expressed as mean ± SEM of three independent experiments and compared using ANOVA. (c,d) ***P < 0.001, compared with normal control group; ^#^P < 0.05, ^##^P < 0.01, ^###^P < 0.001, compared with untreated sepsis group. (e,f) ^###^P < 0.001, compared with sham/control group; **P < 0.01, ***P < 0.001, compared with untreated sepsis group.

The S100A8 and S100A9 protein expression in the lungs were determined by Immunohistochemistry. Narciclasine suppressed the expression of S100A8 and S100A9 in the lungs of neonatal septic rats (Fig. 3a). Plasma levels of S100A8 and S100A9 were measured using ELISA kits. The levels of S100A8 and S100A9 were significantly elevated in the sepsis group without treatment. Narciclasine treatment significantly reduced the plasma levels of S100A8 and S100A9 in neonatal septic rats (Fig. 3b,e). We also estimated the levels of S100A8 and S100A9 in the lung and liver homogenates. Narciclasine treatment showed significant reduction of S100A8 and S100A9 levels in the lung and liver homogenates compared to the untreated sepsis group (Fig. 3c,d,f,g). The S100A8 and S100A9 protein expression in the livers were determined by Immunohistochemistry (Fig. 4a) and Immunoblotting (Fig. 4b). Narciclasine effectively suppressed the S100A8 and S100A9 protein expression in the livers of neonatal septic rats compared to the untreated sepsis group. We also found that narciclasine effectively reduced the S100A8 and S100A9 mRNA expression in the livers when compared to the untreated sepsis group (Fig. 4e,f).

### Narciclasine treatment reduced the liver inflammation and injury in septic neonatal rats

**Figure 5:**
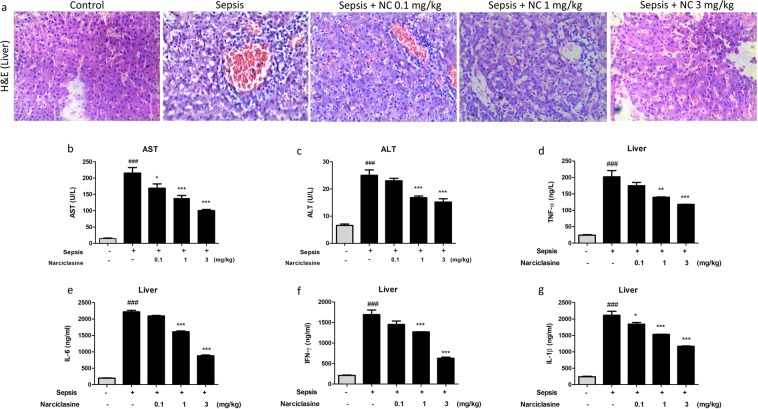
Narciclasine prevented liver damage in neonatal septic rats. The neonatal rats were subjected to *E. coli* sepsis and narciclasine (NC, 0.1, 1 and 3 mg/kg) was administered 1 hour after sepsis induction. Plasma and Livers were collected 12 hours after sepsis induction. (**a**) The liver histological changes in the presence or absence of narciclasine (NC, 0.1, 1, 3 mg/kg) after H&E staining. The original amplification was x200. (**b**) The levels of AST and (**c**) ALT in the plasma were determined using an automated biochemical analyser. (**d**–**g**) The levels of cytokines TNF-α, IL-6, IFN-γ and IL-1β in liver homogenates were determined by ELISA. Data were expressed as mean ± SEM and compared using ANOVA. ^###^P < 0.001, compared with sham/control group; *P < 0.05, **P < 0.01, ***P < 0.001, compared with untreated sepsis group.

The liver is a vital organ and the liver mediated immune response is crucial for eliminating bacteria and toxins in sepsis. Nevertheless in sepsis, the liver is injured by excessive load of pathogens and inflammatory mediators. Liver dysfunction can often occur in the early stages of sepsis. Attenuating the liver injury and restoration of liver function lowers the morbidity and mortality in sepsis^25^. The protective effect of narciclasine on liver was analyzed by histological studies of liver sections stained with haematoxylin and eosin (Fig. 5a). The liver sections from the control rats showed minimal inflammation. The liver sections in untreated sepsis group rats showed increased sinusoidal congestion, edema, inflammation of the portal tract, macrovascicular fatty changes in hepatocytes, central vein congestion and severe infiltration of inflammatory cells of the portal tract. These sepsis induced histological changes in the liver was effectively minimized by narciclasine treatment (1 mg/kg and 3 mg/kg). The liver tissues from narciclasine treated rats showed lesser sinusoidal congestion, edema and inflammatory cell infiltration than the untreated sepsis group rats.

The extent of liver inflammation and injury could also be determined by estimating the levels of liver enzymes aspartate transaminase (AST) and alanine transaminase (ALT) in plasma. The levels of AST and ALT were found to be significantly elevated in the untreated sepsis group. The narciclasine treated group showed significant reduction of AST and ALT compared to untreated sepsis group (Fig. 5b,c).

**Figure 6:**
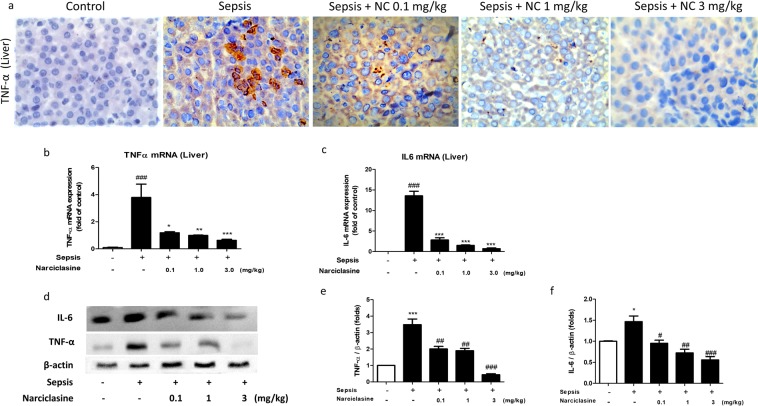
Narciclasine inhibited the expression of TNF-α and IL-6 in the livers of neonatal septic rats. (**a**) The neonatal rats were subjected to *E. coli* sepsis and narciclasine (NC, 0.1, 1 and 3 mg/kg) was administered 1 hour after sepsis induction. Livers were collected 12 hours after sepsis induction. The expression of TNF-α in the liver was determined by Immunohistochemistry. (**b**) The mRNA expression of TNF-α and (**c**) IL-6 in the liver was determined by quantitative real time PCR. (**d**) The protein expression of TNF-α and IL-6 was determined by western blot. (**e**) The relative levels of TNF-α *vs* β-actin and (**f**) IL-6 *vs* β-actin were determined by densitometry. Data were expressed as mean ± SEM of three independent experiments and compared using ANOVA. (b,c) ^###^P < 0.001, compared with sham/control group; *P < 0.05, **P < 0.01, ***P < 0.001, compared with untreated sepsis group; (e,f) *P < 0.05, ***P < 0.001, compared with sham/control group; ^#^P < 0.05, ^##^P < 0.01, ^###^P < 0.001, compared with untreated sepsis group.

We also estimated the levels of inflammatory cytokines – TNF-α, IL-6, IL-1β and IFN-γ in liver homogenates. Narciclasine treatment showed significant reduction of inflammatory cytokines (TNF-α, IL-6, IL-1β and IFN-γ) in liver homogenates compared to the sepsis group without treatment (Fig. 5d–g). The TNF-α protein expression in the liver was determined by Immunohistochemistry. Narciclasine treatment showed significant reduction of TNF-α expression compared to the sepsis group without treatment (Fig. 6a).

We further found that the mRNA expression of TNF-α and IL-6 was found to be significantly increased in the livers of septic neonatal rats without treatment. Narciclasine significantly downregulated the mRNA expression of TNF-α and IL-6 in the liver of septic rats (Fig. 6b,c). Immunoblotting results showed elevated expression of TNF-α and IL-6 in the livers of untreated septic rats. Narciclasine treatment significantly downregulated the protein expression of TNF-α and IL-6 in the livers of septic rats (Fig. 6d–f).

### Narciclasine ameliorated the sepsis induced acute lung inflammation and injury in neonatal rats

**Figure 7:**
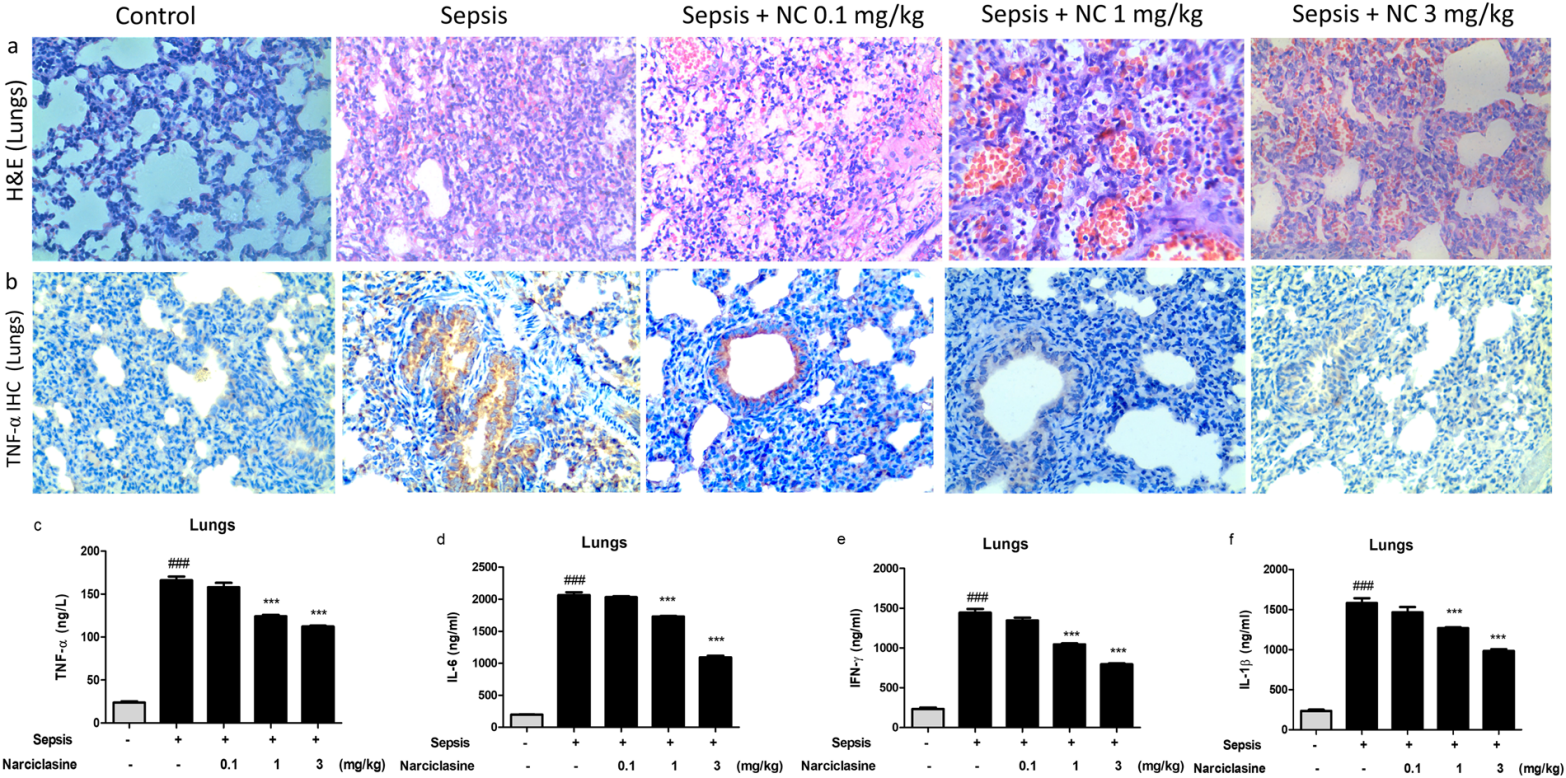
Narciclasine attenuates sepsis induced lung injury and the expression of inflammatory cytokines in lungs of neonatal septic rats. The neonatal rats were subjected to *E. coli* sepsis and narciclasine (NC, 0.1, 1 and 3 mg/kg) was administered 1 hour after sepsis induction. Plasma and Lungs were collected 12 hours after sepsis induction. (**a**) Representative histological sections of lungs collected 12 hours after sepsis induction in rats with and without narciclasine (NC, 0.1, 1 and 3 mg/kg) treatment. Original amplification x200. (**b**) The expression of TNF-α in the lungs was determined by Immunohistochemistry. The results shown are representative of three independent experiments (**c**) The levels of inflammatory cytokines TNF-α, IL-6, IFN-γ and IL-1β in the lung homogenates were determined by ELISA. Data were expressed as mean ± SEM and compared using ANOVA. ^###^P < 0.001, compared with sham/control group; *P < 0.05, **P < 0.01, ***P < 0.001, compared with untreated sepsis group.

Acute lung injury (ALI) is a common complication of sepsis and it occurs due to excessive pulmonary inflammation resulting in high morbidity and mortality^26,27^. The ameliorative effect of narciclasine on lungs was confirmed by histological evaluation of lungs (Fig. 7a). The lung sections in the untreated sepsis group showed severe congestion of interstitial and septal vasculature, increased extravasation of RBCs in alveoli and interstitium, inflammatory cell infiltration, mild edema and emphysematous changes. Narciclasine treatment reduced the intensity of these changes and protected the rats from sepsis induced lung damage. The protein expression of TNF-α in the lungs was determined by Immunohistochemistry. Narciclasine significantly suppressed the expression of TNF-α in the lungs when compared to the sepsis group without treatment (Fig. 7b).

We estimated the concentrations of inflammatory cytokines TNF-α, IL-6, IL-1β and IFN-γ in the lung homogenates (Fig. 7c–f). Treatment with narciclasine significantly reduced the levels of inflammatory cytokines TNF-α, IL-6, IL-1β and IFN-γ in lung homogenates when compared to the sepsis group without treatment. These data show that narciclasine suppresses the lung inflammation and injury in neonatal sepsis.

### Narciclasine reduces the inflammatory anemia in neonatal rats with sepsis

**Figure 8:**
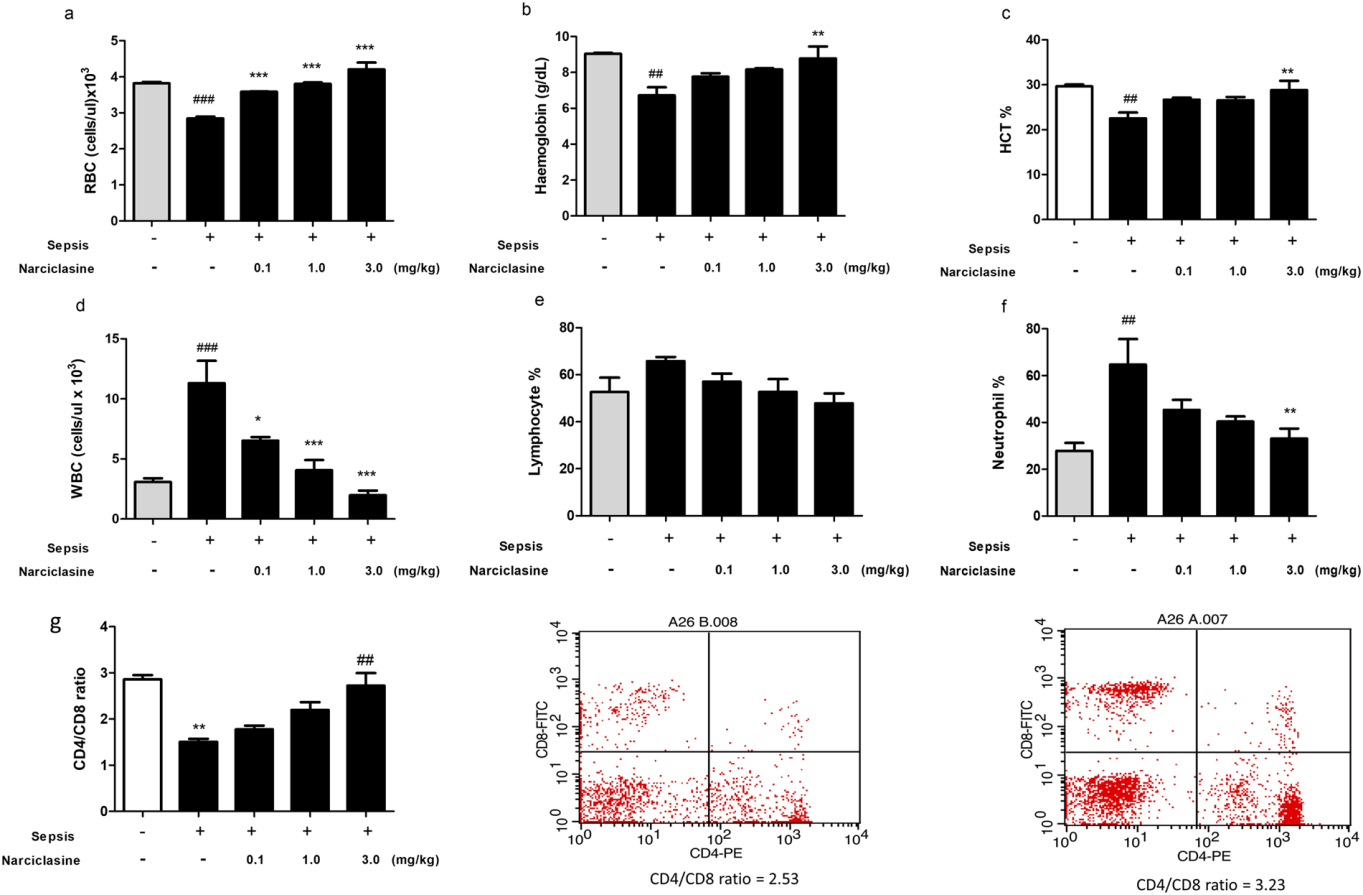
Narciclasine reduced the inflammatory anaemia in neonatal septic rats. The rats were subjected to *E. coli* sepsis (n = 6 per group) in the presence or absence of narciclasine (NC, 0.1, 1 and 3 mg/kg). 12 hours later blood was collected and analysed using an automated haematology analyser. (**a**) Red blood cell count (**b**) Haemoglobin (**c**) Packed cell volume (**d**) White blood cell count (**e**) % Lymphocytes (**f**) % Neutrophils. Data were expressed as mean ± SEM and compared using ANOVA. (**g**) The CD4/CD8 T lymphocyte ratio was determined using flow cytometry. Data were expressed as mean ± SEM and compared using ANOVA. (a–f) ^###^P < 0.001, ^##^P < 0.01, compared with sham/control group; *P < 0.05, **P < 0.01, ***P < 0.001, compared with untreated sepsis group. (g) **P < 0.01, compared with sham/control group; ^##^P < 0.01 compared with untreated sepsis group.

The development of anemia is common during sepsis and highly prevalent in intensive care patients with high risk of sepsis^28^. We analyzed the whole blood parameters which revealed a significant reduction of RBC, hemoglobin and packed cell volume in the untreated sepsis group which was brought to normal levels on narciclasine treatment (Fig. 8 a–c). The WBC count, neutrophil % and lymphocyte % were found to be elevated in the untreated sepsis group. Narciclasine treatment could lower these values to normal levels (Fig. 8d–f). The CD4/CD8 T lymphocyte ratio was also significantly reduced in the sepsis group without treatment. Narciclasine treated rats showed improved CD4/CD8 T lymphocyte ratio (Fig. 8g). These data suggest that narciclasine reduces the inflammatory anemia in neonatal sepsis and improves the outcome.

### Narciclasine inhibited the expression of adaptor proteins of the TLR4 pathway, reduced Iκβα degradation and NF-κβ p65 phosphorylation in livers of neonatal rats with sepsis

**Figure 9:**
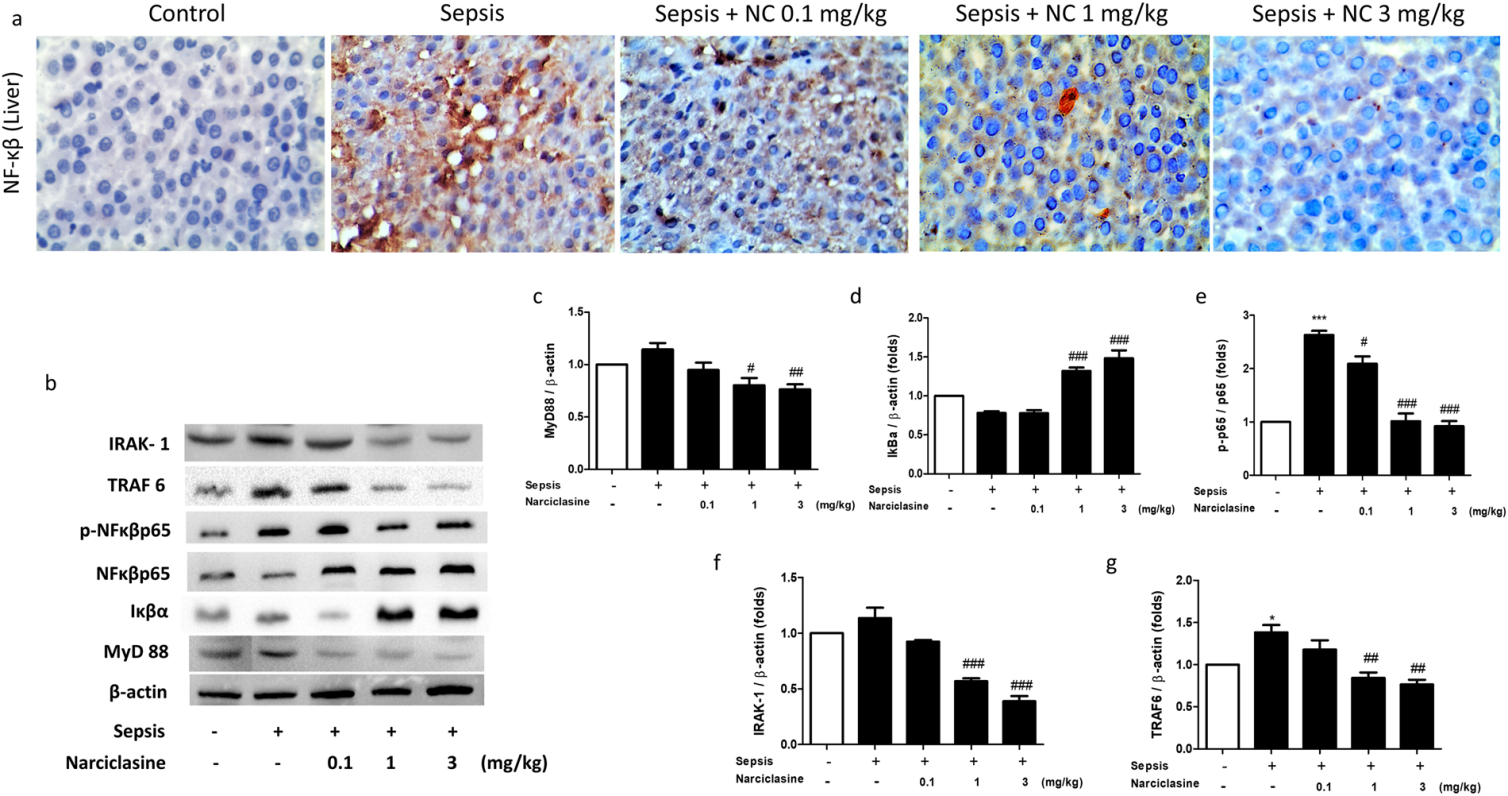
Narciclasine inhibited Iκβ degradation, NF-κβ phosphorylation, MyD88, IRAK-1 and TRAF 6 protein expression in the livers of neonatal rats with sepsis. The neonatal rats were subjected to *E. coli* sepsis and narciclasine (NC, 0.1, 1 and 3 mg/kg) was administered 1 hour after sepsis induction. Livers were collected 12 hours after sepsis induction. (**a**) The protein expression of NF-κβp65 in the livers was determined by Immunohistochemistry (**b**) The expression of Iκβα, NF-κβp65, phosphorylated NF-κβp65, MyD88, IRAK-1 and TRAF-6 were determined by western blot. The relative levels of (**c**) MyD88 *vs* β-actin (**d**) Iκβα *vs* β-actin, (**e**) phospho- NFκβp65 *vs* NF-κβp65, (**f**) IRAK-1 *vs* β-actin and (**g**) TRAF6 *vs* β-actin were determined by Densitometry. Data were expressed as mean ± SEM of three independent experiments and compared using ANOVA. ***P < 0.001, *P < 0.05, compared with sham/control group; ^#^P < 0.05, ^##^P < 0.01, ^###^P < 0.001, compared with untreated sepsis group.

The toll like receptor 4 (TLR-4) pathway has a crucial role in triggering the innate immune response in acute inflammation associated disorders like sepsis^29^. It is activated by bacterial endotoxins which triggers the downstream signaling cascade that results in the production of inflammatory cytokines. Most toll like receptors activate a common intracellular signaling pathway that involve the adaptor proteins myeloid differentiation factor 88 (MyD88), IL-1R associated kinase (IRAK-1) and TNFR-associated factor 6 (TRAF-6)^30,31^. This leads to the nuclear translocation and activation of NF-κβ that subsequently results in production of inflammatory cytokines. In this study, we found that narciclasine suppresses the expression of NF-κβ p65 in the liver tissues of neonatal septic rats as revealed by Immunohistochemical staining (Fig. 9a). Immunoblotting results showed increased NF-κβ p65 phosphorylation in the livers of non-treated septic rats whereas narciclasine treatment significantly suppressed the NF-κβp65 phosphorylation (Fig. 9b). We also found that in comparison to the untreated sepsis group, narciclasine treatment reduced the protein expression of MyD88, IRAK-1 and TRAF-6 in the livers of septic neonatal rats (Fig. 9b). The NF-κβ activation is crucial event in the activation of various inflammatory mediator networks in the pathophysiology of sepsis. The NF-κβ activation is regulated by the Iκβ family of proteins and Iκβs inhibit NF-kappa B activation^32^. We found that the expression of Iκβα in the liver of non-treated sepsis group rats was downregulated whereas narciclasine treatment significantly prevented the Iκβα degradation (Fig. 9b).

## Discussion

Gram negative sepsis is a common clinical syndrome arising due to the severe host response to infection where foreign bacteria and lipopolysaccharide activate the various immune cells^33^. In response to infection, the activation of phagocytes occurs by pathogen associated molecular patterns (PAMPs) which recognize specific pattern recognition receptors (PRRs). The phagocytes can also be activated by endogenous danger signals known as alarmins or damage associated molecular patterns (DAMPs)^34^. The S100A8, S100A9 proteins are considered as important endogenous DAMPs which are abundantly released by phagocytes in response to stress/inflammatory stimuli. S100A8/S100A9 proteins have pro-inflammatory properties which are characterized by the induction of pro-inflammatory cytokines and adhesion molecules in endothelial cells^34,35^. Previous findings have shown that the plasma level of S100A8/S100A9 in neonates with sepsis was found to be highly elevated^8^. The elevated level of S100A8/S100A9 amplifies inflammation and enhances the inflammatory response in infection, making it a potential therapeutic target in anti-inflammatory strategies. In contrast, MRP8/14 (also known as S100A8/A9) is considered important in the innate host defenses against pathogens due to their anti-microbial properties^36,37^. Nevertheless, in fulminant systemic inflammation as induced by high dose of LPS or *E. coli* administration, endogenous MRP8/14 adds to the lethality^38,39^. It has also been found that MRP8/14 complex acts as an endogenous activator of TLR4 and amplifies the lethality in septic shock^39^. The targeted deletion of MRP14 has shown to improve survival in mouse models of LPS induced shock and *E. coli* induced sepsis^39^. This shows the potential influence and effect of the S100A8, S100A9 proteins in conditions like sepsis and shock and the need to neutralize its deleterious effects.

Narciclasine is an isocarbostyril alkaloid of the *Narcissus sp*. and belongs to the Amarylldiaceae family. Previous studies have shown that narciclasine exhibits 250 fold lesser sensitivity on normal lung fibroblasts when compared to cancer cells^40^. Moreover, low concentrations of narciclasine were sufficient to inhibit the cytotoxicity of calprotectin in a rat adjuvant arthritis model^21^. This is one of the significant advantages of using narciclasine which has shown to have significant therapeutic potential in low doses.

Neonatal sepsis is associated with increased levels of inflammatory cytokines which plays a significant part in its pathogenesis and severity^41^. Narciclasine has shown to exhibit anti-inflammatory properties and suggested to be used in inflammation related disorders^20^. Here we observed that *E. coli* sepsis strongly induces the expression of calprotectin and pro-inflammatory cytokines in neonatal rats. This was alleviated by treatment with low concentrations of narciclasine. Previous literature has shown that *E. coli* infection in its fulminant form is characterized by high bacterial load which subsequently can result in circulatory collapse and death within short time span^42^. In our study, we observed that there was reduced bacterial load in the narciclasine treated rats compared to the untreated septic rats. Previous studies have shown that narciclasine does exhibit bacteriostatic activity against *E. coli*^43^. This may be the reason behind the reduced bacterial load in narciclasine treated rats in our study.

Gram-negative sepsis is characterized by tissue and organ injury resulting from the excessive production of cytokines in response to increased amounts of bacteria and LPS^31,44^. Severe sepsis involves the failure of organ systems, commonly known as the multiple organ dysfunction (MOF) syndrome. This generally starts with the failure of respiratory system. This is subsequently followed by the failure of hepatic, cardiovascular, renal, CNS, gastrointestinal and coagulatory system^45^. A large number of neutrophils infiltrate the organs during sepsis and this is associated with impaired organ function and organ failure^46^. The prevention of liver injury and damage has shown to lower the morbidity and mortality in sepsis patients^25^. During sepsis, the activated kupffer cells in liver release inflammatory cytokines such as TNF-α and IL-6 which facilitates multiple organ damage^47^. In our study, the histopathological results showed that narciclasine treatment reduced the liver inflammation and injury. The inflammatory cytokine expression was also suppressed in the liver of narciclasine treated rats. Increase in liver enzymes such as aspartate amino transferase (AST) and alanine aminotransferase (ALT) signify hepatocellular damage in sepsis^48^. In the untreated septic rats we found a drastic rise in the plasma AST and ALT levels which was significantly reduced in the narciclasine treated groups. This shows that narciclasine reduces the intensity of liver damage in neonatal septic rats.

Sepsis and acute lung injury (ALI) have a close relation in the intensive care units and more than 50% of patients with sepsis develop ALI^49^. It was evident from the histopathological findings in our study that narciclasine treatment prevented the lung inflammation and injury. The inflammatory cytokines levels in the lung homogenates were also reduced in the narciclasine treated rats. This suggests that narciclasine has an ameliorative effect on the lungs during neonatal sepsis.

A common complication of severe sepsis is anemia, which is characterized by decrease in the hematocrit and hemoglobin^50^. Erythrocytes are crucial as they deliver oxygen from lungs to tissues. Sepsis is characterized by a reduction of the red blood cell count and a rapid rise in the WBC count^28,51^. We found that narciclasine could normalize the changes in most of these hematological parameters. During sepsis, neutrophils are activated and a large number is released in the blood which in turn releases lytic factors causing local tissue damage^52^. We found that narciclasine treated rats had significantly reduced neutrophil count compared to the untreated septic rats. Furthermore, septic shock is associated with a significant reduction in the CD4/CD8 T cell ratio. This reduction in the lymphocyte subset proportion causes suppressed immune function and enhanced spread of infection in septic shock^53^. In our study we found that narciclasine treatment normalized the CD4/CD8 T lymphocyte ratio in neonatal rats with sepsis.

The pathogenesis of sepsis involves the dysregulated activation of inflammatory mediator networks which results in the hyper-production of inflammatory cytokines. The toll like receptor 4 (TLR-4) pathway is activated by bacterial LPS which is the major component of the cell wall of gram negative bacteria^29^. This subsequently triggers an intracellular signaling cascade that ultimately results in the secretion of inflammatory cytokines. The intracellular and extracellular components of the TLR4 signaling cascade are potential therapeutic targets for the treatment of acute inflammation disorders like sepsis where there is excessive cytokine secretion^29^. The NF-kappa B activation is a central event in the activation of these crucial networks^32^. It has a crucial role in regulating the transcription of immunomodulatory mediators in sepsis and sepsis induced organ failure^54^. The suppression of NF-kappa B activation and phosphorylation has shown to reduce the acute inflammatory processes and organ injury in sepsis. In our study we found that narciclasine could suppress the phosphorylation of NF-κβp65 in the liver. Previous studies have shown that MRP-8 directly interacts with TLR-4/MD-2 and induces NF-κβ activation^39^. MRP8 induces the intracellular translocation of MyD88 and activation of IRAK-1 and NF-κβ that subsequently results in the upregulated expression of TNF-α^39^. In our study we observed that narciclasine reduces the expression of adaptor proteins MyD88, IRAK-1 and NF-κβ p65 which correlate with the reduced expression of S100A8. Thus the protective effect of narciclasine could be mediated by the reduced expression of S100A8 which subsequently downregulates other molecules of the NF-κβ signaling pathway.

Previous literature has shown that narciclasine has shown to exhibit more cytotoxicity towards human cancer cell lines than normal cells^40^. The lower sensitivity of narciclasine towards normal lung fibroblast cells shows that it is less cytotoxic on normal cells. The histopathological studies in our study showed no signs of malignancy in the narciclasine treated rats. Previous studies analyzing the toxicity of narciclasine in wistar rats found no adverse effect at 1 mg/kg/day^55^. This study was done on adult rats and dose given was for 3 weeks. In our study, the dose of 1 mg/kg was found to be optimal and showed significant improvement in outcome with no organ injury or damage in neonatal rats with sepsis. According to the “Conversion of animal doses to human equivalent doses’ of the US FDA this dose is equivalent to 0.162 mg/kg in humans. As this conversion is for rats in general, considering the smaller body surface area and body weight of neonatal rats this value could be lower than 0.162 mg/kg in humans. As the progression of sepsis is rapid and it could cause death within a short span of time, drug compounds which could exhibit significant effect within this short time span could only be of benefit. In our study we used narciclasine at minimum concentrations (0.1, 1 and 3 mg/kg) and found that it could significantly improve the outcome at these minimal concentrations.

These findings establish a novel role and mechanism of action of narciclasine in improving outcome in neonatal septic rats. These results can be further exploited therapeutically for the resolution of sepsis in neonates. Nevertheless, there needs to be further investigations over a longer period of time to assess if there are any side effects associated with these concentrations before human use.

## Materials and Methods

### Reagents and antibodies

Narciclasine was purchased from Tocris Bioscience (Minneapolis, MN, USA). The cytometric bead array (CBA) Rat Inflammation kit was purchased from BD Biosciences, USA. The rat ELISA kits for TNF-α, IL-6, IL-1β and IFN-γ were purchased from Bioassay Technology Laboratory, China. Rat S100A8 and S100A9 ELISA kits were purchased from CusaBio (China). Antibodies against S100A9, NF-κβp65, IL-6 were purchased from Novus Biologicals (Littleton, CO, USA). Antibodies against S100A8 was purchased from Abcam (Cambridge, MA, USA). Antibodies against TNF-α, β-actin, MyD88, IRAK-1 and TRAF-6 were purchased from ABclonal (Woburn, MA, USA). Antibodies against phospho-NFκβp65, Iκβα were purchased from Cell Signaling technology (Danvers, MA, USA).

### Animals

Newborn rat pups along with their mothers were obtained from the Central Animal House, Jawaharlal Institute of Postgraduate Medical Education & Research, Puducherry and were kept in a pathogen free environment under controlled environmental conditions in a 12 h light dark cycle. The study was approved by the Institute Animal Ethics Committee (IAEC), Jawaharlal Institute of Postgraduate Medical Education & Research, Puducherry, India (IAEC 21/6/2015) and was carried out in accordance with the National Institute of Health Guide for the care and use of laboratory animals.

### Pharmacological treatments

Narciclasine was dissolved in DMSO and diluted using saline accordingly to minimize the DMSO concentration to 0.5%. The neonatal rats were randomly divided into the following five groups: Sepsis group without treatment, sepsis treated with narciclasine group receiving 0.1 mg/kg body weight narciclasine; sepsis treated with narciclasine group receiving 1 mg/kg body weight narciclasine; sepsis treated with narciclasine group receiving 3 mg/kg body weight narciclasine. The rats in the control group were administered with an equal amount of vehicle. Narciclasine was administered intraperitoneally 1 hour after induction of sepsis.

### *E. coli* induced sepsis

We used a previously established neonatal rat *Escherichia coli* sepsis model^56,57^. In brief the neonatal rats were intraperitoneally inoculated with 6000~ colony forming units (CFU) of *E. coli* (ATCC 25922) in saline. After 1 hour, narciclasine was administered intraperitoneally to the treatment group neonatal rats at various doses (NC, 0.1 mg/kg, 1 mg/kg and 3 mg/kg). Quantitative cultures were done to check for the levels of bacteremia in blood before treatment and 5 hours after treatment. The rats were also observed for clinical signs of sepsis that includes piloerection, lethargy, increased body temperature, reduced spontaneous activity, huddling and a decrease in food intake^58^. The activity of the rats was monitored using the activity meter (Coulborn Instruments, USA). Rectal temperature was measured periodically to observe for the fluctuations in body temperature.

### Survival study

The neonatal rats were induced with *E. coli* sepsis and after 1 hour administered with narciclasine (NC, 0.1, 1, 3 mg/kg) in various doses. The survival of the rats was monitored for 30 hours. The degree of sepsis was evaluated by the change in body temperature, reduced spontaneous activity, response to touch, decreased feeding, hunched posture and huddling. The clinical signs were scored from 0 to 4. The total score was calculated by adding the individual scores of each parameter. The total score was calculated by adding the individual scores obtained for each parameter adding up to a maximum score of 20.

### Bacterial load

Peritoneal fluid was collected by injection of sterile PBS into the peritoneum and aspirating back. Blood and peritoneal fluid were subjected to serial log fold dilution in sterile saline. Liver and lungs were aseptically collected and homogenized using sterile PBS. Homogenized tissues were briefly centrifuged and the supernatants were subjected to serial log fold dilutions in sterile saline. The bacterial load in blood, peritoneal fluid, liver and lungs was determined by plating 10 fold serial dilutions on tryptic soy blood agar plates. The plates were incubated for 24 h at 37′C and the colonies were counted and expressed as log CFU/ml blood or peritoneal fluid and log CFU/g tissue for organs.

### Quantification of cytokines

For detection of cytokine levels blood was collected 12 hours after sepsis induction into heparinized tubes, through cardiac puncture. Plasma was separated immediately from whole blood by centrifuging at 3000 r.p.m for 15 minutes at 4′C. Multiple soluble cytokines IL-1α, IL-2, IL-4, TNF-α, IL-10 were simultaneously measured by flow cytometry using the cytometric bead array (CBA) Rat Inflammation kit (BD Biosciences, USA). Acquisition was performed using a FACS Calibur II flow cytometer (BD Biosciences, USA). Quantitative results were generated using the FCAP array software. For the measurement of cytokines TNF-α, IL-6, IL-1β and IFN-γ in the organ homogenates, ELISA kits (Bioassay Technology Laboratory, China) were used. Organ homogenates were collected by homogenizing 100 mg of tissue in 1 ml sterile 1X PBS followed by two freeze thaw cycles to break the cell membranes. The homogenates were centrifuged for 5 minutes at 5000 × g at 4′C and the supernatant was aliquoted and stored at −20 °C until analysis.

### Biochemical analysis

Plasma levels of Aspartate amino transferase (AST) and Alanine amino transferase (ALT) expressed as U/l were measured using fully automated biochemical analyser (AU680 Beckman Coulter, USA).

### Hematological analysis

A portion of the blood collected was processed immediately for analyzing blood parameters (Hemoglobin, WBC count, Differential count) using a Sysmex XT-2000i Hematology Analyser (Sysmex Corporation). The CD4/CD8 T lymphocyte ratio was determined by direct immunofluorescence staining method using flow cytometry. In brief, a part of the blood collected from the rats was added to a mixture of fluorochrome-conjugated monoclonal antibodies (APC anti-rat CD3, PE anti-rat CD4, FITC anti-rat CD8α from BD Biosciences, USA) that bind specifically to cell surface antigens. The stained sample was treated with FACS Lysing Solution to lyse erythrocytes in moderate hypotonic setting while conserving the leucocytes. Then the sample was washed to eliminate excess antibody and debris. Finally, the cells were analyzed by flow cytometry (FACS Calibur II, BD Biosciences, USA).

### Haematoxylin and eosin staining of organ sections

Livers and lungs were collected 12 hours after sepsis induction and were fixed in 10% neutral formalin for 24 hours. The fixed organs were paraffin embedded, cut using microtome at 3 µm thickness and stained using Haematoxylin and Eosin (H&E). The histopathological changes were observed using Olympus CX41 microscope equipped with ProgRes CT3 camera (Jenoptik). The sepsis induced changes in the liver such as sinusoidal congestion, inflammatory cell infiltration, central vein congestion, fatty changes were determined in the various groups. Likewise the changes in the lungs such as vascular congestion, necrosis, inflammatory cell infiltration, hemorrhage and interstitial edema were analyzed among the various groups.

### Immunohistochemical analysis

The liver and lung tissues were immediately fixed in 4% paraformaldehyde. They were embedded in paraffin and 3 µm thick sections were cut. For Immunohistochemical staining, EXPOSE Mouse and Rabbit Specific HRP/DAB Detection IHC Kit (Abcam, Cambridge, UK) was used. The protocol given by the manufacturer was strictly followed. In brief, the sections were deparaffinized and the endogenous peroxidase activity was blocked using the hydrogen peroxide block (given along with the kit). The blocked sections were incubated with the primary antibodies. The primary antibodies used were: anti-S100A8 (dilution 1:100, Abcam, Cambridge, MA, USA), anti-S100A9 (dilution 1:100, Novus Biologicals, Littleton, CO, USA), anti-TNFα (dilution 1:100, ABclonal, Woburn, MA, USA), anti-NFκβp65 (dilution 1:100, Novus Biologicals, Littleton, CO, USA). The secondary antibody-HRP conjugate is given along with the kit. Primary antibody binding was visualized using diaminobenzidine (given along with the kit, Abcam). The sections were then counterstained using Mayer Haematoxylin and mounted.

### Quantitative PCR (qPCR)

Total RNA was isolated from the liver tissues using the RNAiso Plus reagent (Takara Bio Inc., Kusatsu, Shiga, Japan) according to the manufacturer’s instructions. Complementary DNA was synthesized from 1 µg total RNA using reverse transcriptase (Takara Bio Inc.). Quantitative real time RT-PCR (qRT-PCR) analysis was performed with the SYBR-RT PCR kit (Takara Bio Inc.) as per the manufacturer’s instructions. Amplification was done in Light cycler (Roche Cobas Z480, Indianapolis, IN, USA) instrument. Primers used for qRT-PCR amplification can be found in Supplementary Table S1. For relative quantification, ∆∆CT comparative threshold cycle (Ct) method, normalized to β-actin mRNA was used.

### Western blot

The livers were excised 12 hours after sepsis induction. The liver tissues were homogenized with ice cold lysis buffer and required amount of protease inhibitors (Sigma-Aldrich, St. Louis, MO, USA). The protein concentration was determined using BCA assay kit (G-Biosciences, MO, USA). A protein load of 50 µg per sample was subjected to 10% or 12% SDS-PAGE (apparatus from Bio-Rad, CA, USA) and electrophoretically transferred to nitrocellulose membranes (Pierce, Thermo Fisher Scientific Inc., MA, USA). The membranes were incubated using 5% nonfat skim milk in TBS with tween20 (TBST) for 1 hour at room temperature. Membranes were then incubated with primary antibodies overnight at 4 °C. After washing, they were incubated with secondary antibodies for 1 h at room temperature. Beta Actin was used as the control for sample loading and integrity. Finally, the membrane was detected using Electro chemiluminiscence method (Pierce, MA, USA) and signals were detected using the Chemidoc XRS + system (Bio-Rad, CA, USA). The densitometry of the bands was quantified using the Image Lab software (Bio-Rad, CA, USA).

### Statistical analysis

The data were expressed as means ± SEM. The statistical analysis was performed using one way analysis of variance followed by Bonferroni test or Kruskal-Wallis followed by Dunn’s test, where appropriate. For mortality tests, the Kaplan-Meir plots were used and statistical analysis by log rank test was performed. Bacterial load results were expressed as medians. P < 0.05 was considered statistically significant. GraphPad Prism ver.5 (GraphPad Software, San Diego, CA, USA) was used for all statistical analyses.

## Supplementary information

Supplementary Information.

## Data Availability

All relevant data supporting the findings of the study are available in this article and its Supplementary Information files, or from the corresponding author upon reasonable request.
