# Supplementary material for: Narciclasine improves outcome in sepsis among neonatal rats via inhibition of calprotectin and alleviating inflammatory responses
Source: Sci Rep. 2020 Feb 19;10:2947. doi: 10.1038/s41598-020-59716-7 (PMC7031385; doi:10.1038/s41598-020-59716-7)
Supplement: Supplementary file 1 — Supplementary Information. [file 41598_2020_59716_MOESM1_ESM.pdf]

Narciclasine improves outcome in sepsis among neonatal rats via inhibition of calprotectin and alleviating inflammatory responses

Manoj Kumar Kingsley[1], Ballambattu Vishnu Bhat *[1], Bhawana Ashok Badhe[2], Benet Bosco Dhas[1], Subhash Chandra Parija [3]

Department of Neonatology[1], Pathology[2] and Microbiology[3], JIPMER, Puducherry, India

SUPPLEMENTARY INFORMATION

*[1]**Corresponding Author**
Ballambattu Vishnu Bhat,
Professor (Paediatrics), Pondicherry Institute of Medical Sciences,
Former HOD, Dept of Neonatology,
JIPMER, Puducherry-605006, India
Email : drvishnubhat@yahoo.com
Mobile: +91 9842351282

Supplementary Table S1. Primers used for qRT-PCR

Name of Gene	Primer	Primer sequence (5’ → 3’)
S100A8	Forward	GGCAACTGAACTGGAGAAGG
	Reverse	CCCACCCTTATCACCAACAC
S100A9	Forward	AGGACCTGGACACAAACCAG
	Reverse	GACTTGGTTGGGCAGATGTT
TNF-α	Forward	ACTCCCAGAAAAGCAAGCAA
	Reverse	CGAGCAGGAATGAGAAGAGG
IL-6	Forward	CCGGAGAGGAGACTTCACAG
	Reverse	ACAGTGCATCATCGCTGTTC
β-actin	Forward	AGCCATGTACGTAGCCATCC
	Reverse	CTCTCAGCTGTGGTGGTGAA

Full Length Western Blots

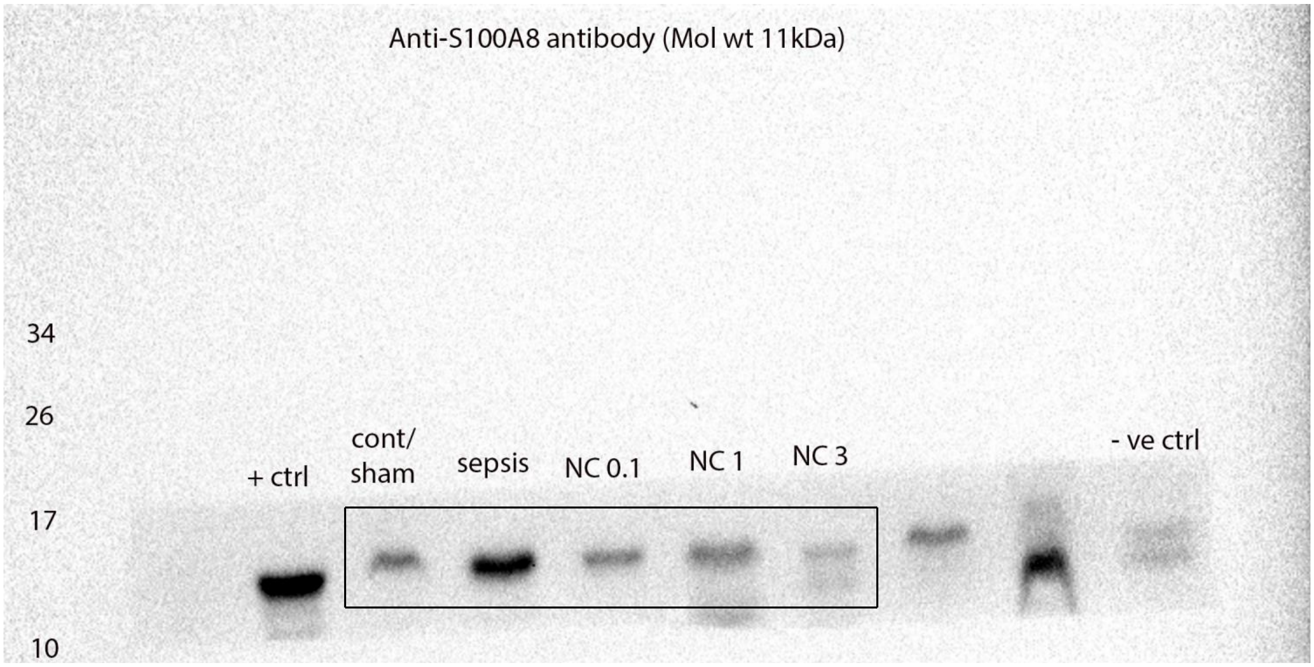

Supplementary Figure S1a shows the full length western blot for S100A8 protein expression in **Fig 4b**. The rectangle box indicates the areas of the western blots shown in the main figure (cont/sham – control group; sepsis group; NC 0.1 – sepsis + narciclasine 0.1 mg/kg group; NC 1 – sepsis+ narciclasine 1 mg/kg group; NC 3- sepsis + narciclasine 3 mg/kg group)

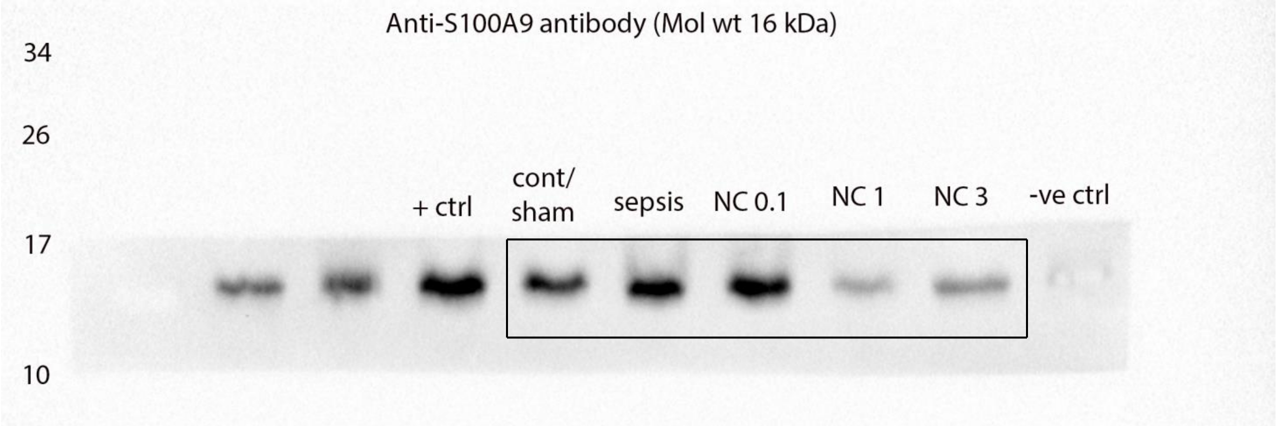

Supplementary Figure S1b shows the full length western blot for S100A9 protein expression in **Fig 4b**. The rectangle box indicates the areas of the western blots shown in the main figure (cont/sham – control group; sepsis group; NC 0.1 – sepsis + narciclasine 0.1 mg/kg group; NC 1 – sepsis+ narciclasine 1 mg/kg group; NC 3- sepsis + narciclasine 3 mg/kg group)

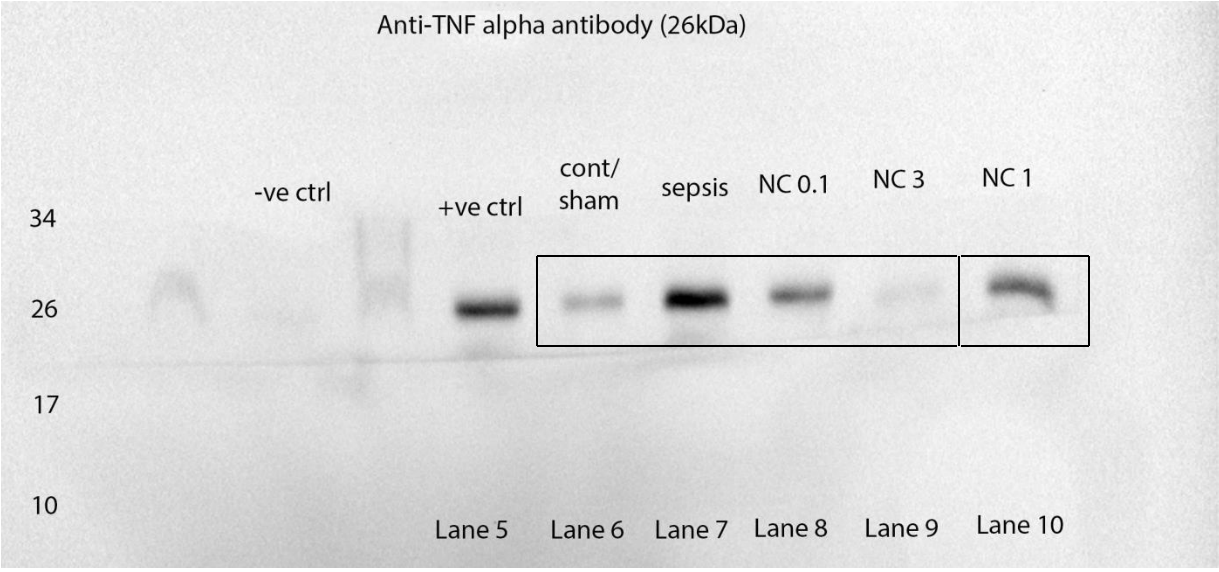

Supplementary Figure S2a shows the full length western blot for TNF-α protein expression in **Fig 6d** of the main manuscript. Note that the lanes 6-9 and tenth lane was cropped out to make a composite image according to the group wise order in **Fig 6d.** The rectangle boxes indicate the areas of the western blots shown in the main figure (cont/sham – control group; sepsis group; NC 0.1 – sepsis + narciclasine 0.1 mg/kg group; NC 1 – sepsis+ narciclasine 1 mg/kg group; NC 3- sepsis + narciclasine 3 mg/kg group)

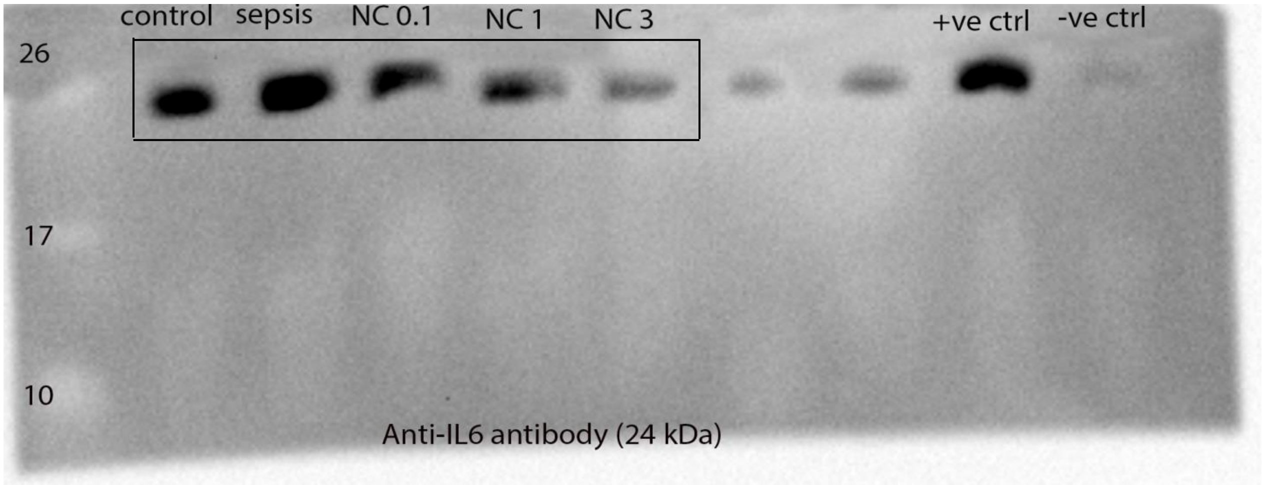

Supplementary Figure S2b shows the full length western blot for IL-6 protein expression in **Fig 6d** of the main manuscript. The rectangle box indicates the areas of the western blots shown in the main figure (control group; sepsis group; NC 0.1 – sepsis + narciclasine 0.1 mg/kg group; NC 1 – sepsis+ narciclasine 1 mg/kg group; NC 3- sepsis + narciclasine 3 mg/kg group)

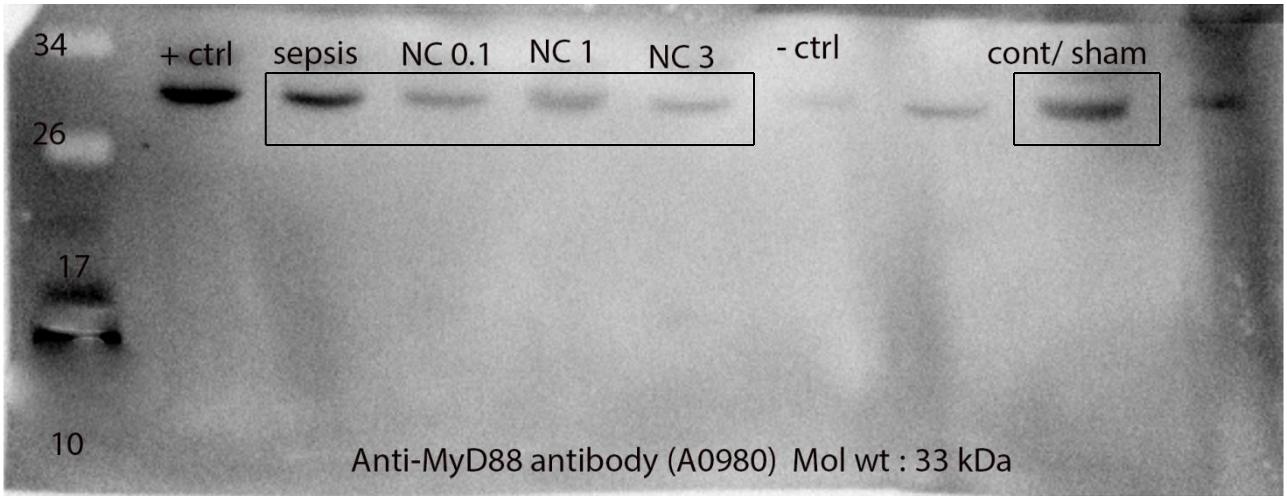

Supplementary Figure S3a shows the full length western blot for MyD88 protein expression in **Fig 9b** of the main manuscript. Note that the lanes 3-6 and ninth lane was cropped out to make a composite image according to the group wise order in **Fig 9b.** The rectangle boxes indicate the areas of the western blots shown in the main figure (cont/sham – control group; sepsis group; NC 0.1 – sepsis + narciclasine 0.1 mg/kg group; NC 1 – sepsis+ narciclasine 1 mg/kg group; NC 3- sepsis + narciclasine 3 mg/kg group)

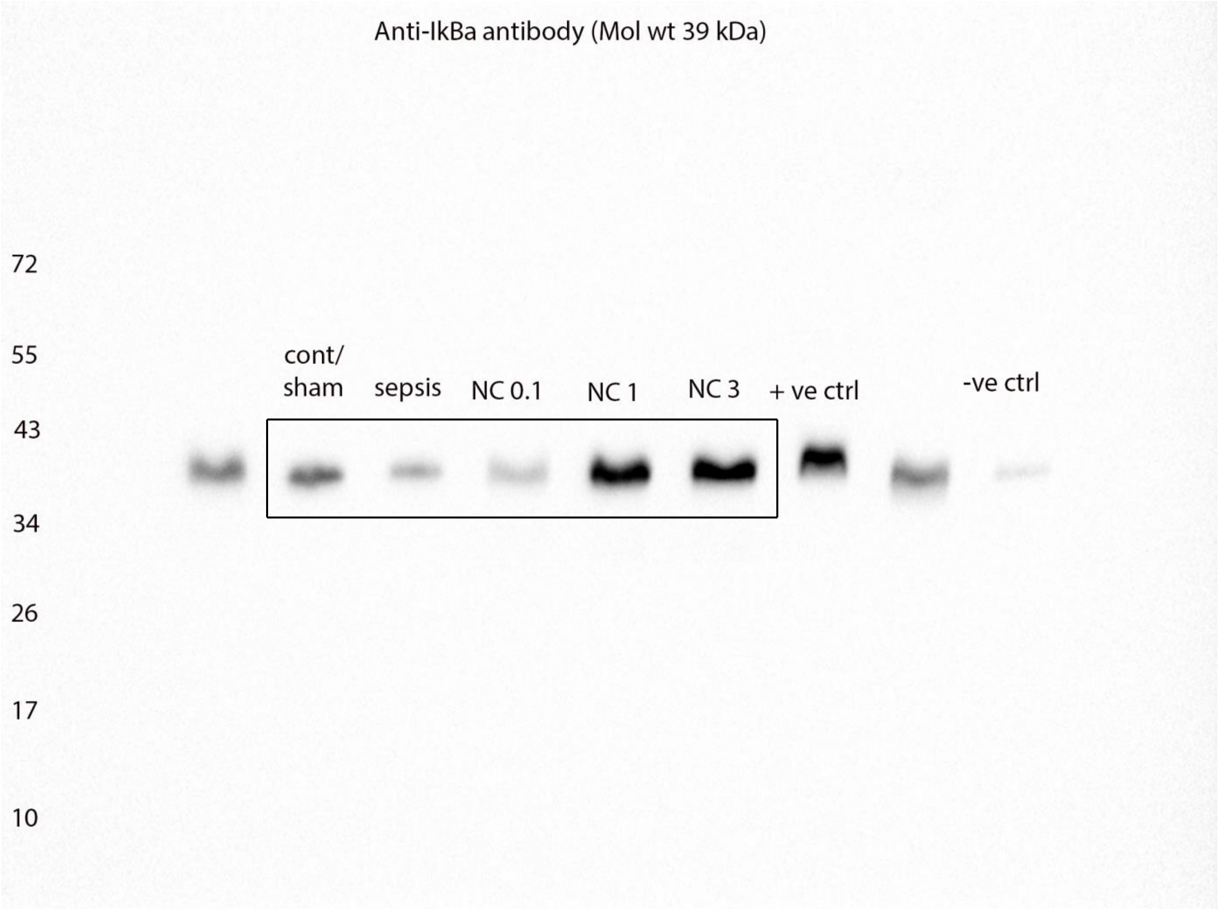

Supplementary Figure S3b shows the full length western blot for Iκβα protein expression in **Fig 9b** of the main manuscript. The rectangle box indicates the areas of the western blots shown in the main figure (cont/sham – control group; sepsis group; NC 0.1 – sepsis + narciclasine 0.1 mg/kg group; NC 1 – sepsis+ narciclasine 1 mg/kg group; NC 3- sepsis + narciclasine 3 mg/kg group)

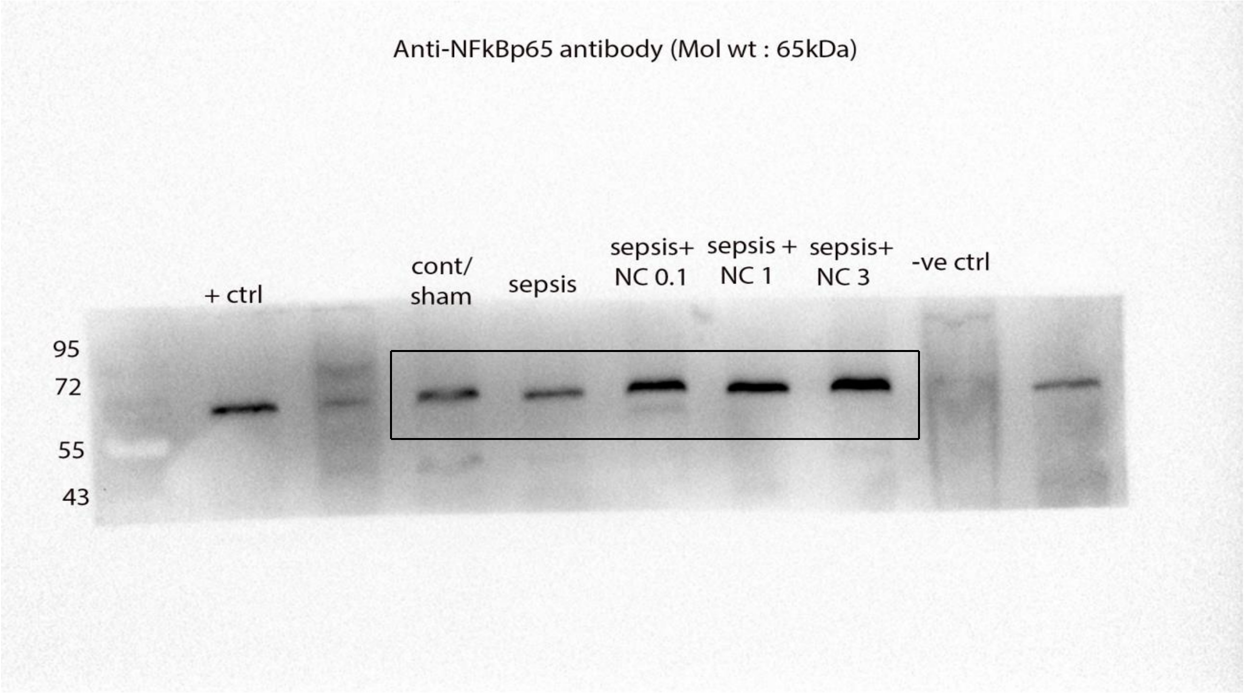

Supplementary Figure S3c shows the full length western blot for NFκβp65 protein expression in **Fig 9b** of the main manuscript. The rectangle box indicates the areas of the western blots shown in the main figure (cont/sham – control group; sepsis group; NC 0.1 – sepsis + narciclasine 0.1 mg/kg group; NC 1 – sepsis+ narciclasine 1 mg/kg group; NC 3- sepsis + narciclasine 3 mg/kg group)

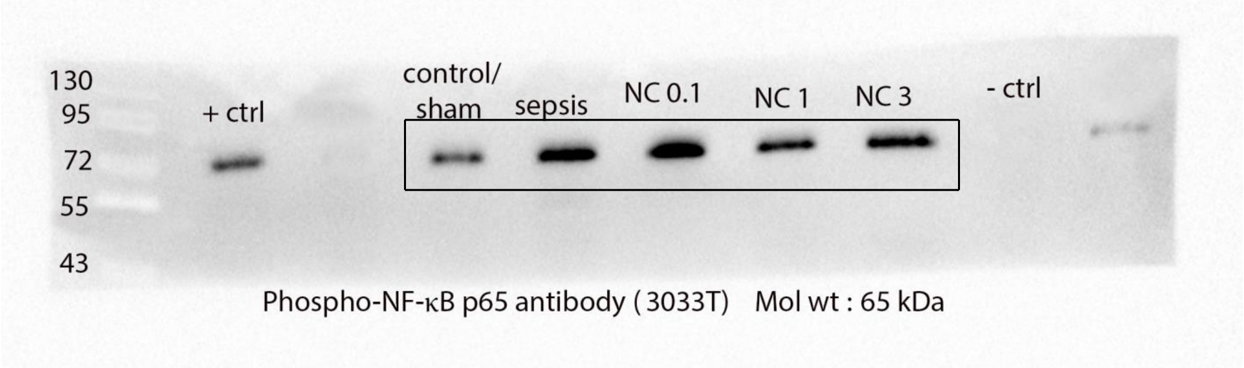

Supplementary Figure S3d shows the full length western blot for phospho-NFκβp65 protein expression in **Fig 9b** of the main manuscript. The rectangle box indicates the areas of the western blots shown in the main figure (control/sham – control group; sepsis group; NC 0.1 – sepsis + narciclasine 0.1 mg/kg group; NC 1 – sepsis+ narciclasine 1 mg/kg group; NC 3- sepsis + narciclasine 3 mg/kg group)

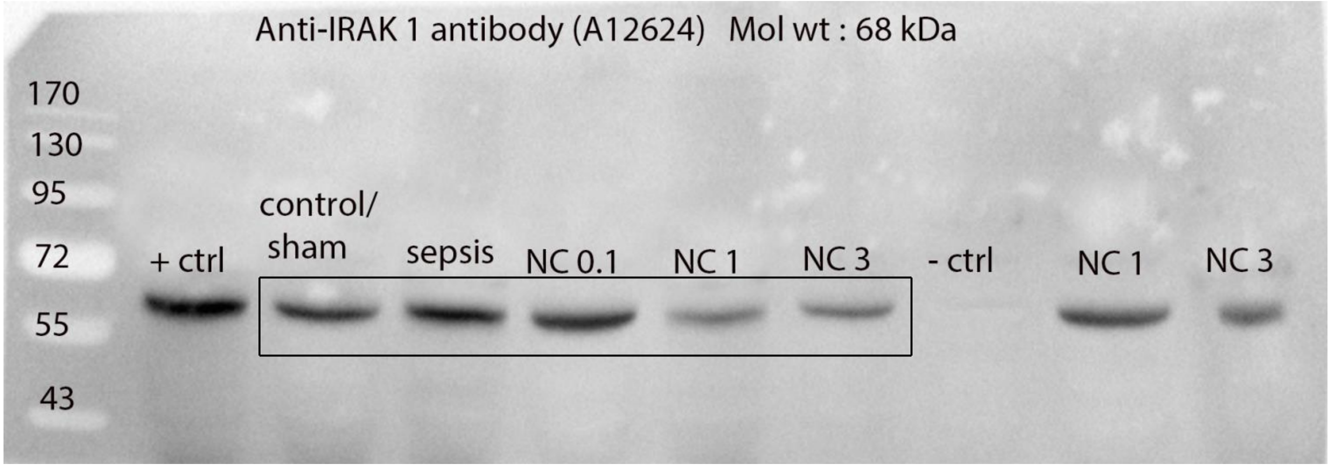

Supplementary Figure S3e shows the full length western blot for IRAK-1 protein expression in **Fig 9b** of the main manuscript. The rectangle box indicates the areas of the western blots shown in the main figure (cont/sham – control group; sepsis group; NC 0.1 – sepsis + narciclasine 0.1 mg/kg group; NC 1 – sepsis+ narciclasine 1 mg/kg group; NC 3- sepsis + narciclasine 3 mg/kg group)

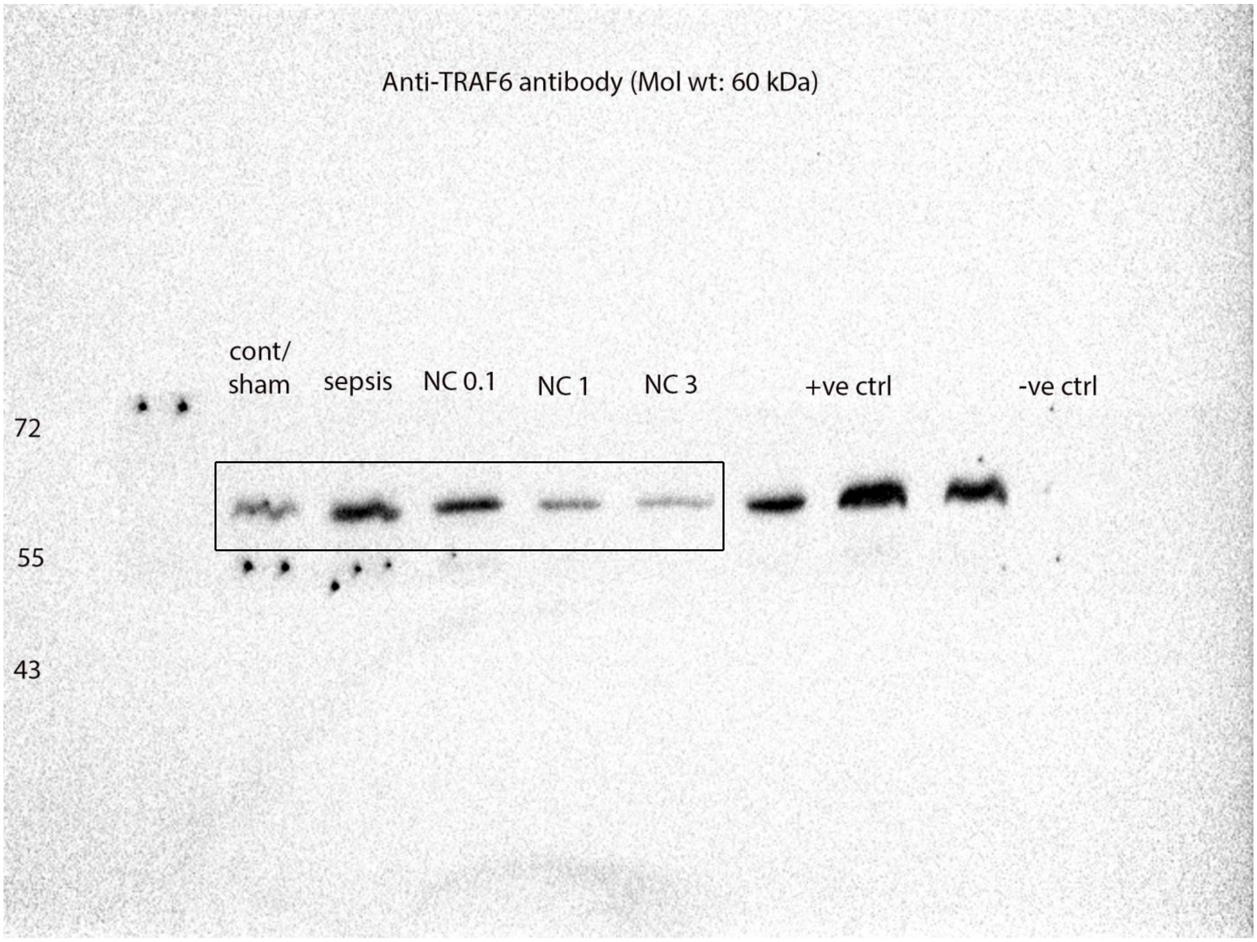

Supplementary Figure S3f shows the full length western blot image for TRAF 6 protein expression in **Fig 9b** of the main manuscript. The rectangle box indicates the areas of the western blots shown in the main figure (cont/sham – control group; sepsis group; NC 0.1 – sepsis + narciclasine 0.1 mg/kg group; NC 1 – sepsis+ narciclasine 1 mg/kg group; NC 3- sepsis + narciclasine 3 mg/kg group)
